# Distinct profiles of size-fractionated iron-binding ligands between the eastern and western subarctic Pacific

**DOI:** 10.1038/s41598-021-81536-6

**Published:** 2021-01-21

**Authors:** Yoshiko Kondo, Rise Bamba, Hajime Obata, Jun Nishioka, Shigenobu Takeda

**Affiliations:** 1 Graduate School of Fisheries and Environmental Sciences, Nagasaki University, Nagasaki, Japan; 2 Organization for Marine Science and Technology, Nagasaki University, Nagasaki, Japan; 3 Pan-Okhotsk Research Center, Institute of Low Temperature Science, Hokkaido University, Sapporo, Japan; 4 Graduate School of Environmental Science, Hokkaido University, Sapporo, Japan; 5 Atmosphere and Ocean Research Institute, The University of Tokyo, Kashiwa, Japan

**Keywords:** Environmental sciences, Ocean sciences

## Abstract

Iron (Fe) is well known as a limiting factor to control primary productivity especially in high-nutrient and low chlorophyll area such as the subarctic Pacific. The solubility of Fe is believed to be controlled by its complexation with natural organic ligands, while the distribution of organic ligands is poorly understood. Here, we report that dissolved (< 0.2 µm) organic ligands were unevenly distributed between the western and eastern stations in the subarctic Pacific. The concentration of dissolved organic ligands around the lower part of subarctic Pacific intermediate water was higher in the western station, suggesting that Fe complexation with these organic ligands supports a lateral transport within the water mass. However, a more detailed size-fractionated treatment indicated no significant difference in the soluble (< 1000 kDa) ligands’ distribution between the western and eastern stations. These results suggest that organic and inorganic colloid formations are potentially essential for Fe transport mechanisms in the subarctic Pacific.

## Introduction

**Figure 1:**
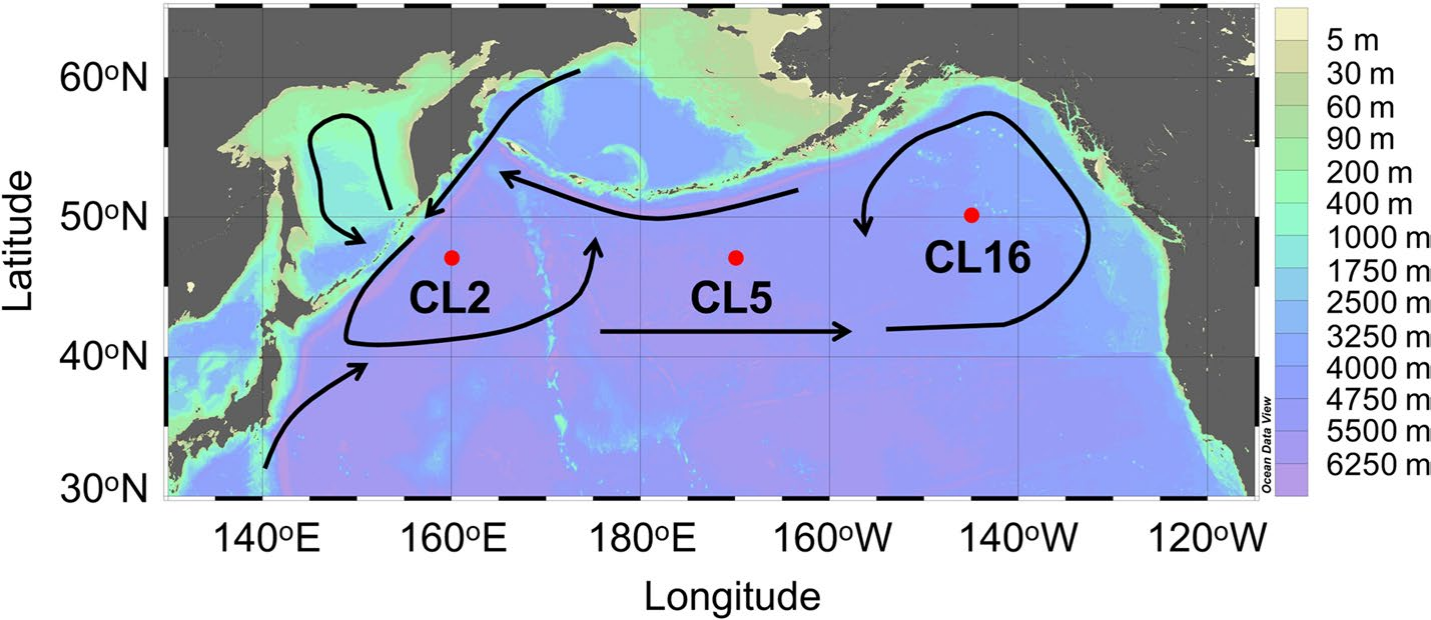
Sampling locations during R/V *Hakuho Maru* KH-17-3 cruise in this study.

Trace metals affect oceanic primary productivity, and their biogeochemical cycling has been researched extensively over the past decade and a half. Iron (Fe) controls approximately 30% of oceanic primary productivity^1^. Therefore, the Fe cycle's precise role in the marine environment has become the subject of great interest and research. In seawater, dissolved Fe is predominantly complexed with a heterogeneous natural organic ligands pool^2^, which regulates both the dissolved Fe concentration ([D-Fe]) and its bioavailability for phytoplankton^3^. Broad groupings of exopolymer substances, humic substances, and siderophores typically act as natural organic ligands in seawater^4^. The subarctic Pacific is well-known as a high-nutrient and low chlorophyll (HNLC) region. The growth of natural phytoplankton is limited by Fe bioavailability^5^. In this area, two cyclonic gyres, the western subarctic gyre (WSG) west of 175°E and the Alaskan gyre (AG) east of 165°W, dominate circulation (Fig. 1). Recent studies demonstrated a gradient in [D-Fe] of intermediate water, which has the same density range as North Pacific Intermediate Water (NPIW, σ_θ_ = 26.6–27.5), located in the North Pacific subarctic gyre^6,7^. The NPIW density range in the subarctic Pacific is significantly influenced by water from the Okhotsk Sea in the upper part (σ_θ_ = 26.6–27.0) and the East Kamchatka Current in the lower part (σ_θ_ = 27.0–27.5)^8^. Therefore, it has been suggested that Fe-rich waters in the western subarctic Pacific are exported from the Okhotsk and Bering Seas^7^. Furthermore, a fluorometric study has demonstrated that refractory shelf humic substances from the Okhotsk Sea transports dissolved Fe to the subtropical North Pacific via the NPIW^9^. The difference in Fe speciation between east and west was also indicated by the comparison of the Fe(III) hydroxide solubility^10^. In the western North Pacific, higher [D-Fe] compared with the Fe(III) hydroxide solubility has been observed in the deep-water column, suggesting the higher production of dissolved Fe from decomposition of sinking particulate organic matter^10^. As an important factor controlling the [D-Fe] in the ocean, determining natural organic ligands' distributions, including humic substances, may reflect the specific features in this area. Therefore, this study investigated the vertical size-fractionated distributions of organic Fe-binding ligands in the eastern and western subarctic Pacific to establish their impact on the Fe cycle.

## Results and discussion

### Hydrography of the study area

Stations CL2, CL5, and CL16 were located in the WSG, central North Pacific (CNP), and the AG (Fig. 1). Generally, the subarctic Pacific is characterized by low salinity surface waters that result in significant stratification between surface waters and intermediate waters^6^. In surface water, concentrations of nutrients and chlorophyll *a* in the western station were relatively higher than those in the eastern station (Supplementary Fig. S1, Table S1), consistent with the previous observation^6^. During our observation, the subarctic intermediate water was distributed from 150 to 1250 m depths (σ_θ_ = 26.6–27.5) (Supplementary Table S1). A rapid decrease in the dissolved oxygen (DO) and increase in the apparent oxygen utilization (AOU) was observed below the surface mixed layer. In general, the concentration of nutrients below the surface mixed layer reflected the regeneration of organic matters (Supplementary Fig. S1, Table S1). At Stn. CL2, relatively high concentration of nutrients was observed in the upper NPIW compared to the other stations. Considering phosphate ([PO_4_^3−^]) as an example for nutrients, the regenerated and preformed phosphate could be evaluated as follows:

$$\begin{aligned} & {\left[ {{\text{P}}{{\text{O}}_4}^{3 - }} \right]_{{\text{Regenerated}}}} = {\text{ AOU}}\,\left( {{\text{observed}}} \right) \times \left( {1/170} \right),\,{\text{and}} \\ & {\left[ {{\text{P}}{{\text{O}}_4}^{3 - }} \right]_{{\text{Preformed}}}} = \, \left[ {{\text{P}}{{\text{O}}_4}^{3 - }} \right] \, \left( {{\text{observed}}} \right) - {\left[ {{{\text{PO}}_4}^{3 - }} \right]_{{\text{Regenerated}}}} \\ \end{aligned}$$

where the fixed value (1/170) is the constant stoichiometric Redfield ratio between phosphorus production and oxygen consumption^11^; the [PO_4_^3−^]_Regenerated_ follows the same trend as that of AOU (Supplementary Fig. S1). In the upper NPIW density range at Stn. CL2, the [PO_4_^3−^]_Preformed_ (1.36–1.42 μM) was similar to that at Stn. CL5, but slightly higher than that at Stn. CL16 (1.19–1.33 μM). There was not clear difference in [PO_4_^3−^]_Preformed_ between sampling stations in the lower NPIW density range. These results suggested that the preformed phosphate in the upper NPIW, which included transported phosphate from the Okhotsk Sea, may have been higher in the west compared to the eastern region during our observation period.

### Fe and its speciation in the dissolved fraction (< 0.2 μm)

**Figure 2:**
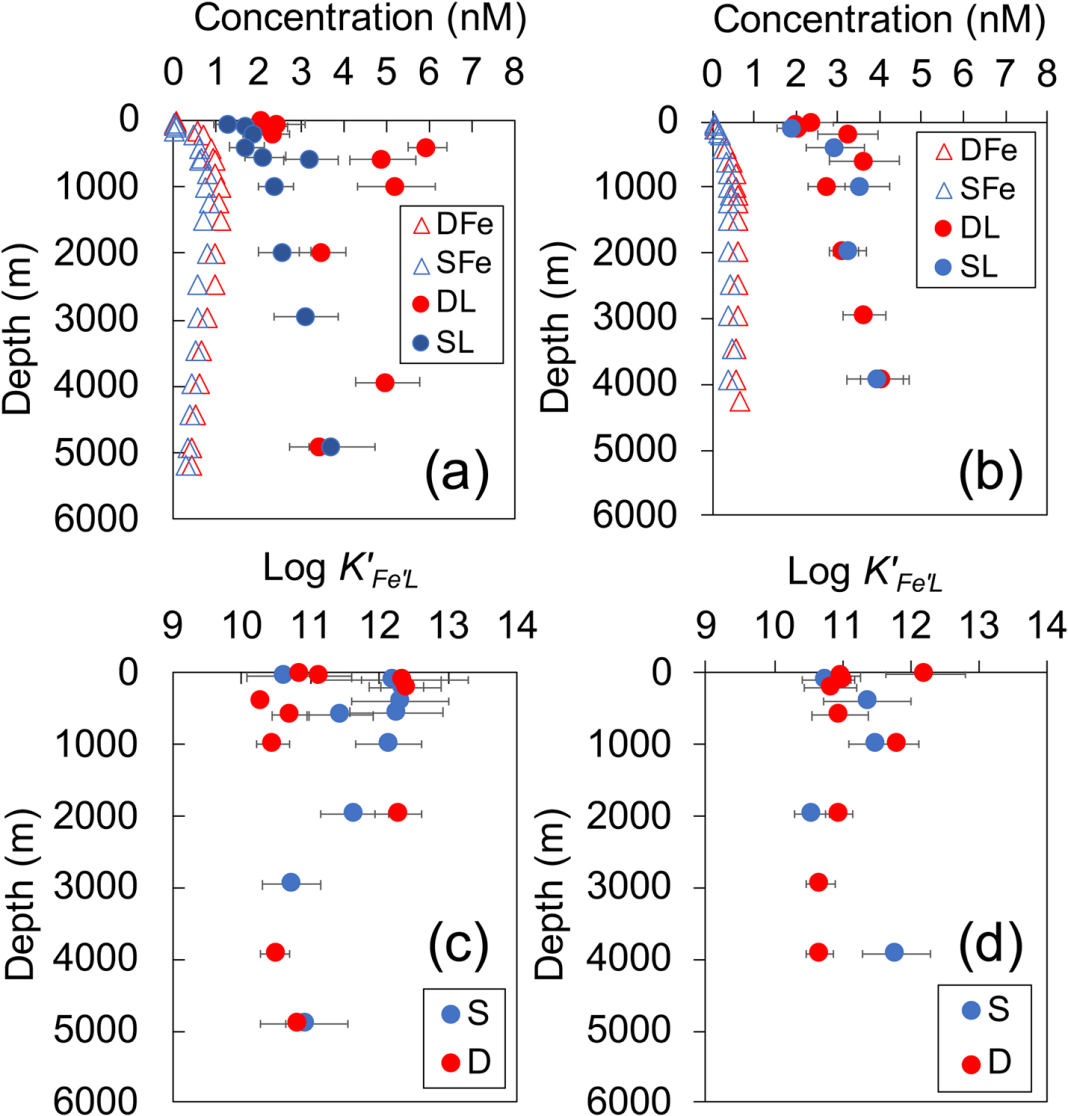
Vertical distributions of [D-Fe] (DFe, red open triangle), [S-Fe] (SFe, blue open triangle), [D-L] (DL, red closed circle) and [S-L] (SL, blue closed circle) at Stns. (**a**) CL2 and (**b**) CL16. Vertical distributions of the *K*′_*Fe*′*L*_ (in log scale) of dissolved (D, red closed circle) and soluble (S, blue closed circle) fractions at Stns. (**c**) CL2 and (**d**) CL16. Error bars indicate analytical error from ProMCC calculation.

**Figure 3:**
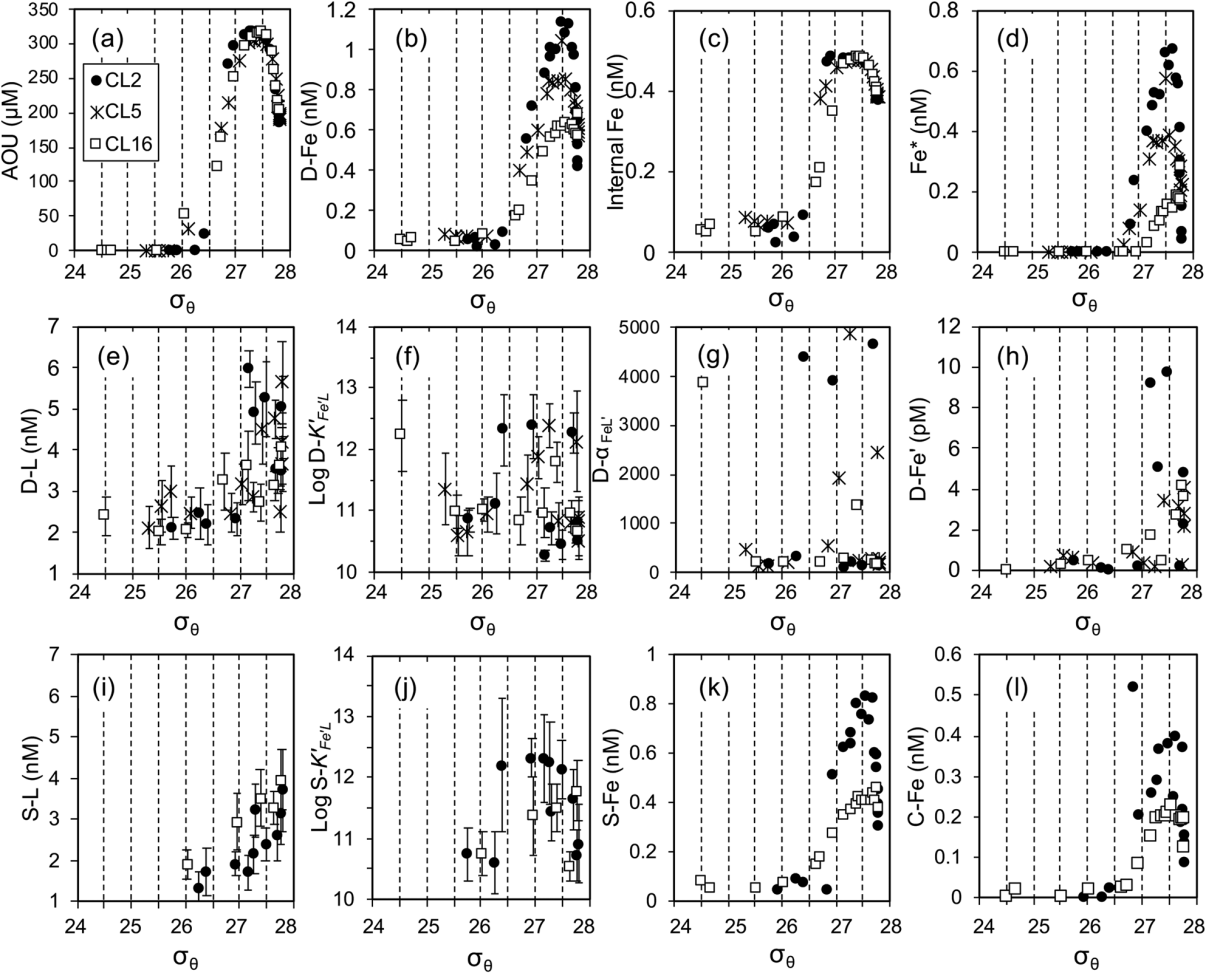
Relationships between σ_θ_ and observed parameters ((**a**)–(**l**), see text) at Stns. CL2 (closed circle), CL5 (asterisk) and CL16 (open square). Error bars in (**e**), (**f**), (**i**) and (**j**) indicate analytical error from ProMCC calculation.

In the upper 100 m at all three stations, the [D-Fe] ranged from 0.02 to 0.09 nM as low as Fe limitation for phytoplankton growth could occur (Fig. 2, Supplementary Table S1). An increase in depth from 100 m resulted in a corresponding increase in [D-Fe], which reached a maximum toward the lower NPIW density range (1000–1250 m). The highest [D-Fe] was recorded at Stn. CL2 in the WSG. Below the maximum layer, [D-Fe] gradually decreased with depth at Stns. CL2 and CL5. However, this trend was not observed at Stn. CL16 (Fig. 2 and Supplementary Fig. S1). Generally, the vertical profile of dissolved Fe below the surface mixed layer reflects the influence of external suppliers such as atmospheric Fe and laterally transported D-Fe (external Fe). Internally regenerated D-Fe (hereafter internal Fe) from processes such as remineralization and desorption from organic particles in the water column are also reflected in this vertical profile^6^. The previous study^6^ applied the Fe* concept^12^ to distinguish between external Fe and internal Fe as follows:

$$\begin{aligned} & {\text{Fe}}^{*} = \left[ {{\text{external}}\,{\text{Fe}}} \right] = \left[ {{\text{D-Fe}}} \right]\,\left( {{\text{observed}}} \right) - \left( {\left[ {{\text{P}}{{\text{O}}_4}^{3 - }} \right]\,\left( {{\text{observed}}} \right) \times {{\text{R}}_{{\text{Fe}}:{\text{P}}}}} \right),\,{\text{and}} \\ & \left[ {{\text{Internal}}\,{\text{Fe}}} \right] = \left[ {{\text{D-Fe}}} \right]\,\left( {{\text{observed}}} \right) - {\text{Fe}}^{*} \\ \end{aligned}$$

where R_Fe:P_ is the ratio of [D-Fe] to [PO_4_^3−^]^6^. In this study, the fixed R_Fe:P_ value (0.16 nM/μM) was applied to determine the relative values Fe* and [Internal Fe] which in turn helped to compare the influence that lateral transport has on [D-Fe] between the sampling locations; the fixed R_Fe:P_ value was the average value of the intermediate water data from Stn. CL16, where the intermediate water may not be strongly influenced by lateral external Fe input^6^. The relationship between σ_θ_, AOU, [internal Fe], and Fe* indicated that dissolved Fe regeneration started to occur below the upper NPIW (Fig. 3). By contrast, an increase in Fe* occurred prominently near the lower NPIW density range, suggesting that the Fe-rich western water in the NPIW can be explained by external Fe sources^6^. The Fe* at Stn. CL16 was lower throughout the water column than those in the western stations, resulting in a different vertical [D-Fe] profile shape for this station.

In this study, dissolved organic ligand concentration ([D-L]) and its conditional stability constant (D-*K*′_*Fe*′*L*_) ranged from 2.01 to 5.96 nM and 10^10.5^ to 10^12.4^ M^−1^, respectively (Figs. 2 and 3, Supplementary Table S1). The [D-L] exceeded [D-Fe] by more than 1.6 nM in all the samples. The range of concentration of inorganic Fe in the dissolved phase ([D-Fe′]), which was not complexed with dissolved organic ligands (see “Methods” section), was 0.01 to 10 pM. Consequently, > 99.3% of dissolved Fe was estimated to be complexed with these natural organic ligands. High excess ligand concentrations (> 3.9 nM) with low D-*K*′_*Fe*′*L*_ (< 10^11.0^) were detected in the lower NPIW density range at Stn. CL2. The complexation capacity of organic Fe-binding ligands (α_FeL′_), which was calculated from the product of the concentration of excess ligands ([L′], see “Methods” section) and *K*′_*Fe*′*L*_, for dissolved fraction (D-α_FeL′_) in the water column ranged from 95 to 4850, within the reported values range (one class ligand, 0-501187)^13^. At Stn. CL2, relatively low D-α_FeL′_ (95–200) was found in the lower NPIW density range, resulting in high [D-Fe′] (Fig. 3). This suggests that excess ligands in the water mass do not contribute directly to increasing [D-Fe]. However, this trend was not observed at Stns. CL5 and CL16.

Previous studies have also reported excess ligands in the Pacific Ocean water column^14–26^. An excess of [D-Fe] relative to [D-L] has been demonstrated in deep waters (> 2000 m)^18,20^, suggesting a variation in dissolved Fe speciation between the Pacific Ocean’s upper and deep waters. This variation is possibly the result of the difference in the competitive ligand exchange-adsorptive cathodic stripping voltammetry (CLE-ACSV) method used in the studies. The previous studies^18,20^ used 2-(2-thiazolylazo)-*p*-cresol (TAC) as the competing ligand instead of salicylaldoxime (SA) used in this study (see “Methods” section). TAC is also recognized as one of the common competing ligands used to determine organic Fe-binding ligands in seawater^27,28^. However, it was suggested that using the CLE-ACSV method with TAC is inefficient for detecting Fe complexation with humic substances^29^. Indeed, dissimilarity in the CLE-ACSV method results has been reported for deep water in the Pacific Ocean^16^ and the Arctic Ocean^30^. Generally, the CLE-ACSV method using 10 μM of TAC reagent underestimates ligand concentrations.

### Fe and its speciation in the soluble and colloidal fractions

The concentrations of both soluble and colloidal Fe ([S-Fe] and [C-Fe], respectively) were generally low in the surface waters. The [C-Fe] was calculated by subtracting the actual measured [S-Fe] from [D-Fe] (see “Methods” section); [S-Fe] should not exceed [D-Fe]. Although slightly exceeding [S-Fe] compared to [D-Fe] was often observed in the upper 50-m of the water column at Stns. CL2 and CL16 (Supplementary Table S1), the excess values were likely negligible because both [S-Fe] and [D-Fe] concentrations were near the detection limit. Therefore, our result suggests that most of the dissolved Fe in the surface water was in the soluble phase. Both [S-Fe] and [C-Fe] increased with depth in the NPIW density range, these concentrations were generally higher at the western station, similar to the behavior of [D-Fe] (Figs. 2 and 3, Supplementary Table S1). By contrast, the vertical profiles of the ligand concentration and *K*′_*Fe*′*L*_ in soluble phase ([S-L] and S-*K*′_*Fe*′*L*_, respectively) were similar for the western and eastern stations; [S-L] gradually increased with depth from the surface to the bottom layer, and the average S-*K*′_*Fe*′*L*_ in the water column was 10^11.4 ± 0.7^ M^−1^ (n = 16). The soluble α_FeL′_ (S-α_FeL′_) in all samples ranged from 49 to 2942, similar to that of the dissolved fraction. The concentration of inorganic Fe in soluble fraction ([S-Fe′]) ranged from 0.03 to 4.4 pM, relatively lower than those in the dissolved fraction. These results indicate that most of the soluble Fe is complexed with these natural organic ligands similar to the dissolved Fe. By contrast, the estimated colloidal α_FeL′_ (C-α_FeL′_), where S-α_FeL′_ was subtracted from D-α_FeL′_ (see “Methods” section), ranged from a negative value to 3876 (Supplementary Table S1). The negative value was observed in the samples from the lower NPIW density range at Stn. CL2 and in the deep water at Stn. CL16.

On an average, 73% of the dissolved Fe was partitioned into the soluble fraction throughout the water column at Stns. CL2 and CL16 (Supplementary Table S1). For the organic ligands, the ratio of the complexation capacity between the soluble and dissolved fractions (S-α_FeL′_/D-α_FeL′_) showed a broad range (16 to > 100%) (Fig. S2). Particularly for the samples from the lower NPIW density range at Stn. CL2, S-α_FeL′_ overwhelmed D-α_FeL′,_ indicating that there were no colloidal ligands with a capacity to bind new Fe in these samples (Supplementary Table S1). The saturation of organic ligands by Fe in the colloidal fraction was also detected in samples from the Southern Ocean^31^ and the Atlantic Ocean^32,33^. Although details such as the reagent type and the applied detection window in the CLE-ACSV method were different for these studies, several parts of colloidal ligands might not be detected by our method.

This study applied a relatively low detection window to increase the peak current’s sensitivity^21^. Therefore, it is possible that the added Fe in the CLE-ACSV titration was absorbed onto the natural inorganic Fe colloids instead of SA in the sample during the equilibration. If so, this would result in an overestimation of the ligand concentration. Further research is required to validate the colloidal ligand concentrations obtained using the CLE-ACSV method.

### Relationship between Fe and organic ligands dynamics in the western subarctic Pacific

Our results revealed that [D-L] always exceeded [D-Fe], indicating an abundant supply of dissolved organic ligands for dissolved Fe in the subarctic Pacific. Moreover, an approximately proportional relationship existed between Fe and ligand concentrations in the dissolved fraction. However, this trend was not observed in the soluble phase (Supplementary Fig. S3). A proportional relationship between α_FeL′_ and the Fe concentration was only observed below the subsurface water (> 100 m) except for the dissolved fraction of the lower NPIW density range at Stn. CL2. The ratio of S-α_FeL′_/D-α_FeL′_ has been introduced to evaluate whether the size-fractionation of Fe ([S-Fe]/[D-Fe]) is controlled by ligands’ behavior^33^. There was no clear relationship between the size-partitioning dissolved Fe and the ligands’ complexation capacity for the study area (Supplementary Fig. S2), consistent with the results from North Atlantic samples^33^. In this study, an anomalously high S-α_FeL′_/D-α_FeL′_ ratio was obtained in the samples from the lower NPIW density range at Stn. CL2, reflecting the low D-α_FeL′_. These results suggested that α_FeL′_ alone cannot explain the dissolved and soluble Fe distributions in the North Pacific and the North Atlantic.

**Figure 4:**
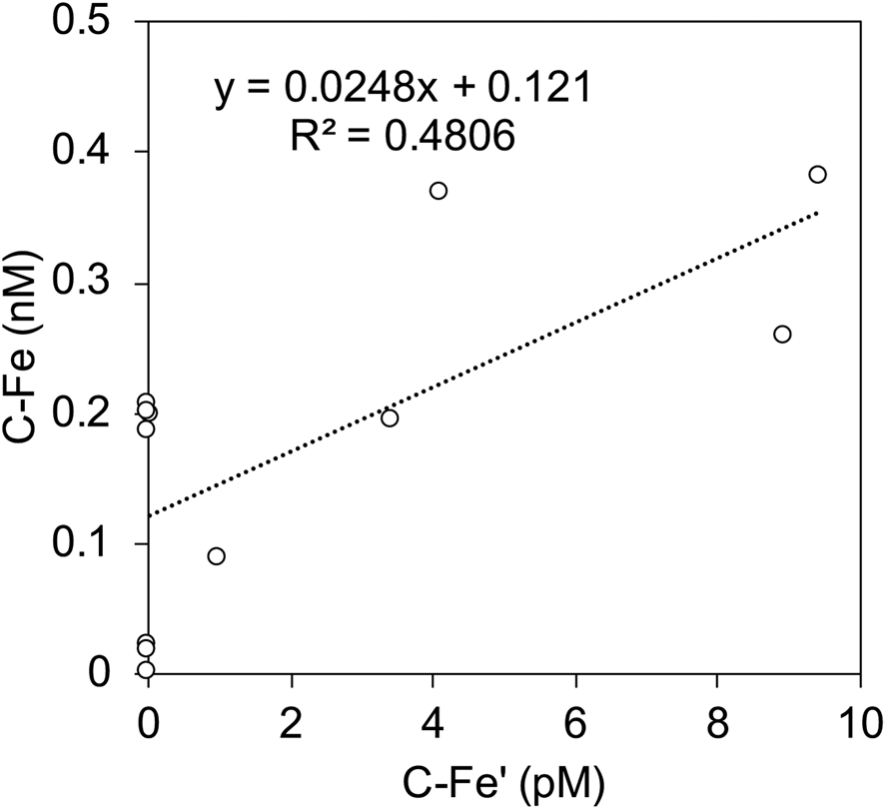
Relationship between [C-Fe′] and [C-Fe] at Stns. CL2 and CL16 in this study.

As mentioned above, our method may have miscalculated several portions of organic ligands. As a result, the negative value of C-α_FeL′_ in the lower NPIW density range at Stn. CL2 was calculated, indicating the existence of “not organically complexed” Fe in the colloidal fraction. The concentration of colloidal inorganic Fe ([C-Fe′]) was calculated from the equation [C-Fe′] = [D-Fe′]–[S-Fe′] to evaluate Fe speciation in the colloidal fraction. Interestingly, there was a positive correlation between [C-Fe′] and [C-Fe] in this study area (Fig. 4). Particularly, high [C-Fe′] and [C-Fe] were observed in the lower NPIW density range at Stn. CL2, suggesting that colloidal Fe in the lower NPIW density range at Stn. CL2 contained an inorganic colloidal form and organic complexes that were not detectable by our CLE-ACSV method. Furthermore, the behavior of [D-Fe′] was partially similar to Fe* (Fig. 3), suggesting that external Fe tended to form unstable colloidal Fe in the western region. These findings are consistent to the previous study which demonstrated higher [D-Fe] over the solubility of Fe(III) hydroxide (in this case, < 0.025 μm fraction) in the deep-water column of the western Pacific (165°E)^10^.

**Figure 5:**
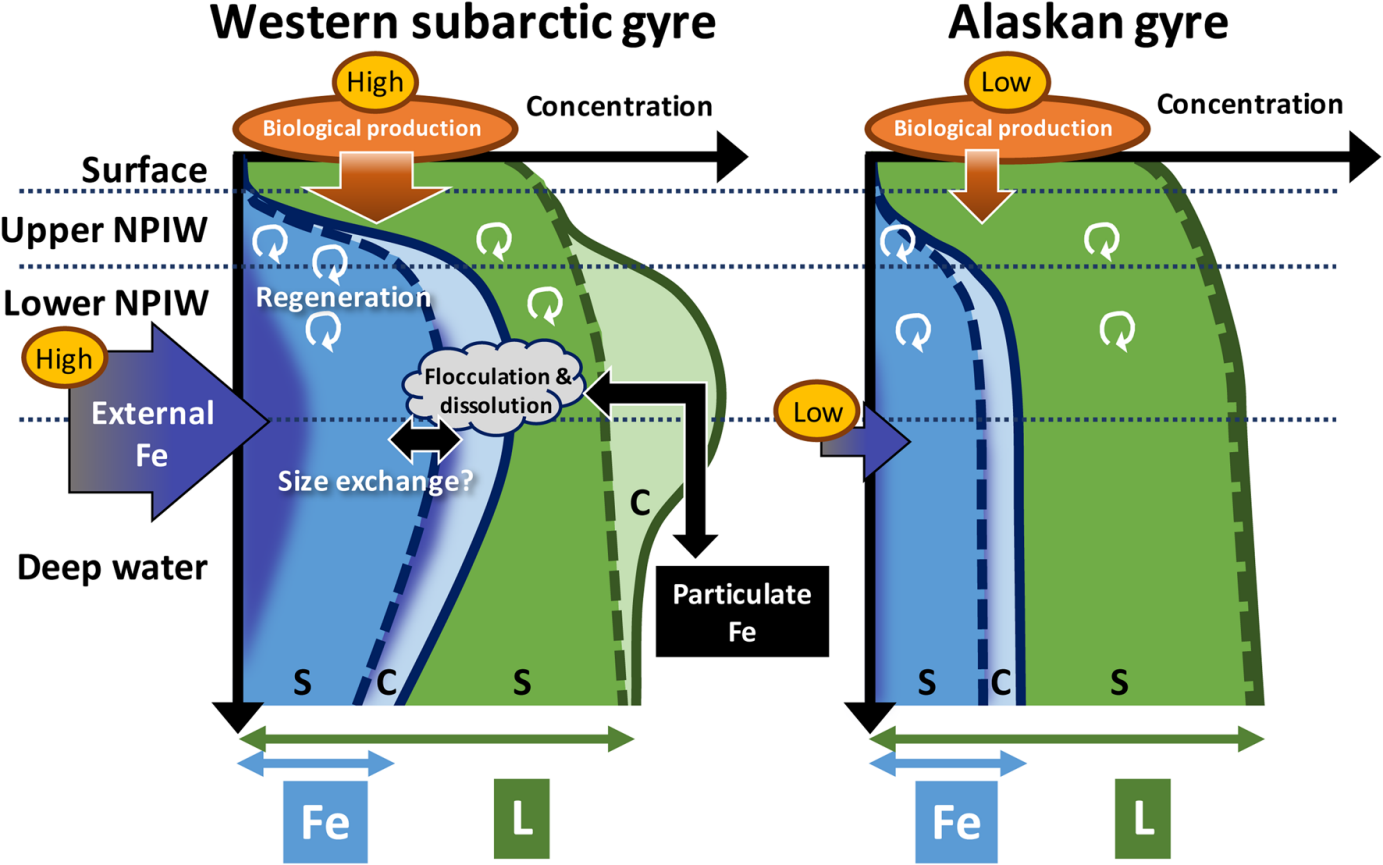
Schematic of the differences in the vertical distributions of size-fractionated Fe speciation between the Western subarctic gyre (WSG) and the Alaskan gyre (AG). Light blue and blue color areas indicate the Fe concentration. Light green and green color areas indicate the excess ligands concentration. Dashed lines indicate the boundary between the soluble (S) and colloidal (C) fractions. Blue purple color areas in Fe concentrations indicate the influence of external Fe. The dotted lines indicate the boundaries between water masses in this study. In the WSG, higher external Fe input (blue purple arrow) from the Okhotsk Sea (mainly upper NPIW) and the East Kamchatka Current (mainly lower NPIW) was expected to the intermediate water, as well as decomposition of sinking particulate organic matter from the surface water (orange arrow). These processes also supply the organic ligands to the water column. Theoretically, there are sufficient organic ligands, which can complex with Fe throughout the water column in both areas. However, below the lower NPIW in the WSG, there were unstable inorganic Fe and organic complexes that were not detectable by our CLE-ACSV method^21^. The co-existence of unstable colloidal Fe and excess ligands suggests the potential of reversible exchange of Fe mediated by colloids in the WSG (black arrows) (see text).

Judging from the Fe behaviors in the western subarctic Pacific, the east–west differences in the biological production and lateral transport via NPIW formation are essential factors that control Fe distribution and chemical speciation (Fig. 5). It has been suggested that high Fe supplies via vertical mixing with intermediate waters from the Okhotsk and Bering Seas and dust input results in high productivity in the western subarctic Pacific compared to the eastern area^7,34^. The distribution of organic ligands could be influenced by multiple biological sources including the release of extracellular polymeric substances by phytoplankton, and by degradation process such as photochemical reaction in the surface water^4,28^. As a result, the Fe speciation in the surface water was similar between Stns. CL2 and CL16 during our observation period despite the east–west differences in biological productivity^34^. The high productivity in the upper waters would cause a high flux of sinking particles and partially contribute to the high regeneration of macro- and micro-nutrients, including Fe and its organic ligands. In this study, the rapid increases of AOU and [PO_4_^3−^]_preformed_ were observed in the upper NPIW at Stn. CL2 (Supplementary Fig. S1). It was demonstrated that humic substance-like fluorescent dissolved organic matter, which includes some part of Fe-binding organic ligands, supported the transportation of dissolved Fe and nutrients via the intermediate water from the Okhotsk Sea^9^, indicating the relatively high [D-Fe] in the upper NPIW at Stn. CL2 was derived from the Okhotsk Sea. In the lower NPIW density range, high Fe* and [D-L] were observed in the western station, suggesting an external source of Fe and organic ligands from the Bering Sea. Since some parts of these external Fe might exist as inorganic colloidal forms and organic complexes that were not detectable by our CLE-ACSV method^21^, the unstable colloidal Fe would be partially flocculated and scavenged by sinking particles in the deep water. However, the existence of excess ligands in both size fractions throughout the water column also indicated the potential dissolution of particulate Fe via complexation, suggesting the reversible exchange of Fe mediated by colloids between the soluble, colloidal and particulate phases, especially near the lower NPIW. Taken together, further research incorporating a multiple analytical windows analysis for the CLE-ACSV^22^ is still required to clarify the detail of colloidal ligands’ behavior and its influence on size-exchange of Fe in this area. However, our results suggest that dissolved Fe speciation in the NPIW density range in the western subarctic Pacific has unique features relative to those in the eastern area. Considering that the NPIW density range sources are derived from the Okhotsk Sea and the East Kamchatka Current, further high-resolution observation is required to clarify the source of organic ligands and the relationship between Fe speciation and the transport mechanism in the western subarctic Pacific.

## Methods

### Sample collection and treatment

Seawater samples were obtained from three stations in the subarctic Pacific Ocean onboard R/V *Hakuho Maru* KH-17-3 (GEOTRACES GP02) cruise between June 23 and August 7 in 2017. Samples were collected using acid-cleaned Teflon coated 12-L Niskin-X bottles attached to a CTD-Carousel Sampling system suspended on a Vectran cable (Fig. 1). After the recovery of Niskin-X bottles, these bottles were placed in a clean-air booth. Seawater samples were filtered through an acid-clean AcroPak 200 Capsule filter with a 0.2 μm pore-size Supor Membrane (Pall) attached directly to the spigot with silicon tubing. Compressed clean air was then used to obtain the dissolved Fe concentrations of the samples. The seawater samples from Stns. CL2 and CL16, which were measured for soluble Fe and natural organic Fe(III)-binding ligands, were additionally filtered using an acid-cleaned polyethylene hollow fiber filter (< 1000 kDa)^35^. These filtered seawaters were collected in acid-cleaned 500-mL fluorinated high-density polyethylene bottles (Nalgene) and low-density polyethylene bottles (Nalgene) for the analyses of organic Fe-binding ligands and Fe concentrations, respectively. Samples collected for organic ligand analysis were stored at –20 °C until analysis. Fe concentration analysis samples were acidified to pH < 1.7 with 20% quartz-distilled HCl (Tamapure AA-100, Tama Chemicals).

### Fe concentrations

The [D-Fe] and [S-Fe] were determined onboard with an automatic Fe(III) flow injection analytical system (Kimoto Electric Co. Ltd.). This system was used with a chelating resin preconcentration and the Chemiluminescence Detection method^7,36^. A 10 M formic acid-2.4 M ammonium buffer solution was added to the samples. The samples were then adjusted to pH 3.2 using 20% NH_4_OH (Tamapure AA-100; Tama Chemicals) immediately before analysis. The [C-Fe] was calculated from [C-Fe] = [D-Fe]–[S-Fe]. The Fe analysis detection limit was 0.038 nM. SAFe reference standards S1, D1, and D2^37^ were measured during the sample analysis. The results of S1, D1, and D2 were 0.098 ± 0.013 nM, 0.653 ± 0.067 nM, and 0.944 ± 0.059 nM, respectively. These values are within the consensus values (GEOTRACES website).

### Organic Fe-binding ligands

Natural Fe-complexing organic ligand concentrations and their *K*′_*Fe*′*L*_ were determined by CLE-ACSV using SA and an air-pressurized system for mercury drop formation^21^. This method’s details are referenced in the literature^2,21^. In this study, standard Fe additions of 0, 0.25, 0.5, 0.75, 1, 1.5, 2.5, 3.5, 5, 7.5, 10, and 12.5 nM were used in the titrations. The equilibration period occurred overnight (at least 6 h) after the addition of 5 μM SA. The concentrations of dissolved and soluble ligands and their *K*′_*Fe*′*L*_ were then calculated using the ProMCC software with a non-linear fitting^38^. This study used the detection window at 5 μM SA corresponding to α′_Fe′SA_ of 17.9 (side reaction coefficient α_Fe_′ = 10^10^, log *K*′_*Fe*′*SA*_ = 6.52, log *β*′_*Fe(SA)2*_ = 10.72)^21^. Only one class ligand was detected using this detection window. The complexation capacity of natural organic Fe-binding ligands (α_FeL′_) for dissolved and soluble fractions (D-α_FeL′_ and S-α_FeL′_, respectively) was calculated from α_FeL′_ = [L′] × *K*′_*Fe*′*L*_. [L′] indicates the concentration of excess ligands that were not complexed with Fe. Because *K*′_*Fe*′*L*_ occasionally differs between the dissolved and soluble fractions, we did not evaluate the ligand concentration in the colloidal fraction. Instead, we calculated α_FeL′_ in the colloidal fraction (C-α_FeL′_) from C-α_FeL′_ = D-α_FeL′_—S-α_FeL′_ to evaluate the capacity of colloidal ligands’ complexation. Furthermore, the concentration of inorganic Fe ([Fe′]), which was not complexed with natural organic ligands, was estimated from concentrations of Fe and organic Fe-binding ligands, and the *K*′_*Fe*′*L*_ in each fraction (D-Fe′ and S-Fe′).

### Other parameters

Concentrations of chlorophyll *a* and nutrients (NO_3_^−^ + NO_2_^−^, PO_4_^3−^ and SiO_2_) were also sampled and measured on-board. Seawater samples for chlorophyll *a* analysis were immediately filtered through GF/F filters (Whatman). Chlorophyll *a* was extracted in 6-mL aliquots of N, N-dimethylformamide, stored at − 20 °C for over 24 h, and analyzed using a fluorometer (10-AU, Turner Design Inc.). For nutrient analysis, seawater was collected into a 10-mL polyethylene tube. The nutrients were determined by a continuous flow system (SWAAT, BLTEC Japan). DO data were obtained from a CTD sensor. The DO concentration was calibrated using automatic titrator data (DOT-15X, Kimoto Electric Co.). AOU was calculated from the dissolved oxygen, temperature, and salinity using the program Ocean Data View (https://odv.awi.de/).

## Data availability

The datasets presented in the current study are available from supplementary Table S1. All [D-Fe] data in this study is cited from Nishioka et al.^7^ to calculate organic ligands data (https://www.pnas.org/content/117/23/12665).

## Supplementary information

Supplementary Figures.

Supplementary Tables.
