# Supplementary material for: Distinct profiles of size-fractionated iron-binding ligands between the eastern and western subarctic Pacific
Source: Sci Rep. 2021 Jan 21;11:2053. doi: 10.1038/s41598-021-81536-6 (PMC7820233; doi:10.1038/s41598-021-81536-6)
Supplement: Supplementary file 1 — Supplementary Figures. [file 41598_2021_81536_MOESM1_ESM.pdf]

Supplementary figures for

Distinct profiles of size-fractionated iron-binding ligands between the eastern and western subarctic Pacific

Yoshiko Kondo[1,2*], Rise Bamba[3,4], Hajime Obata[5], Jun Nishioka[3], Shigenobu Takeda[1]

[1] Graduate School of Fisheries and Environmental Sciences, Nagasaki University

[2] Organization for Marine Science and Technology, Nagasaki University

[3] Pan-Okhotsk Research Center, Institute of Low Temperature Science, Hokkaido University

[4] Graduate School of Environmental Science, Hokkaido University

[5] Atmosphere and Ocean Research Institute, The University of Tokyo

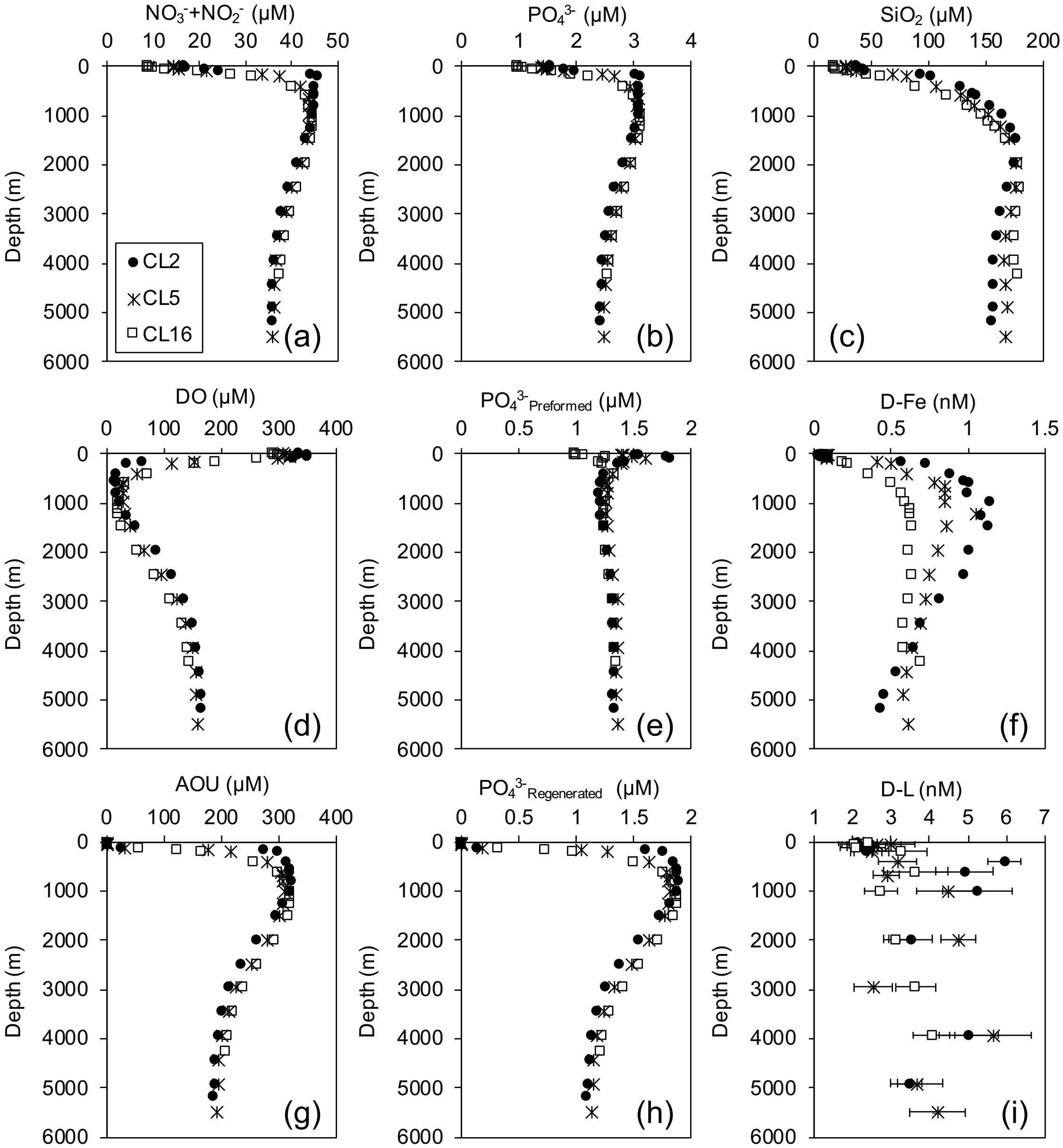

Fig. S1. Vertical distributions of (a) NO_3^-+NO_2^-, (b) PO_4^{3-}, (c) SiO_2, (d) DO, (e) Preformed PO_4^{3-}, (f) D-Fe, (g) AOU, (h) Regenerated PO_4^{3-}, and (i) D-L concentrations at Stns. CL2 (closed circle), CL5 (asterisk) and CL16 (open square). The method to calculate preformed and regenerated PO_4^{3-} is shown in the text.

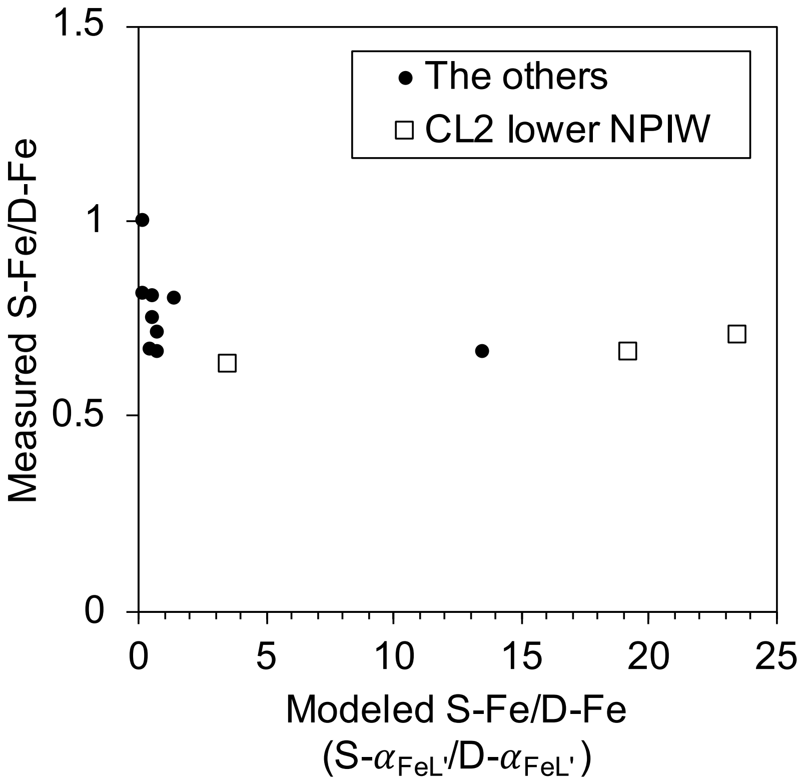

Fig. S2. Relationship between modeled S-Fe/D-Fe (S-$\alpha_{FeL'}$/D-$\alpha_{FeL'}$) and measured S-Fe/D-Fe at Stns. CL2 and CL16 (see the text).

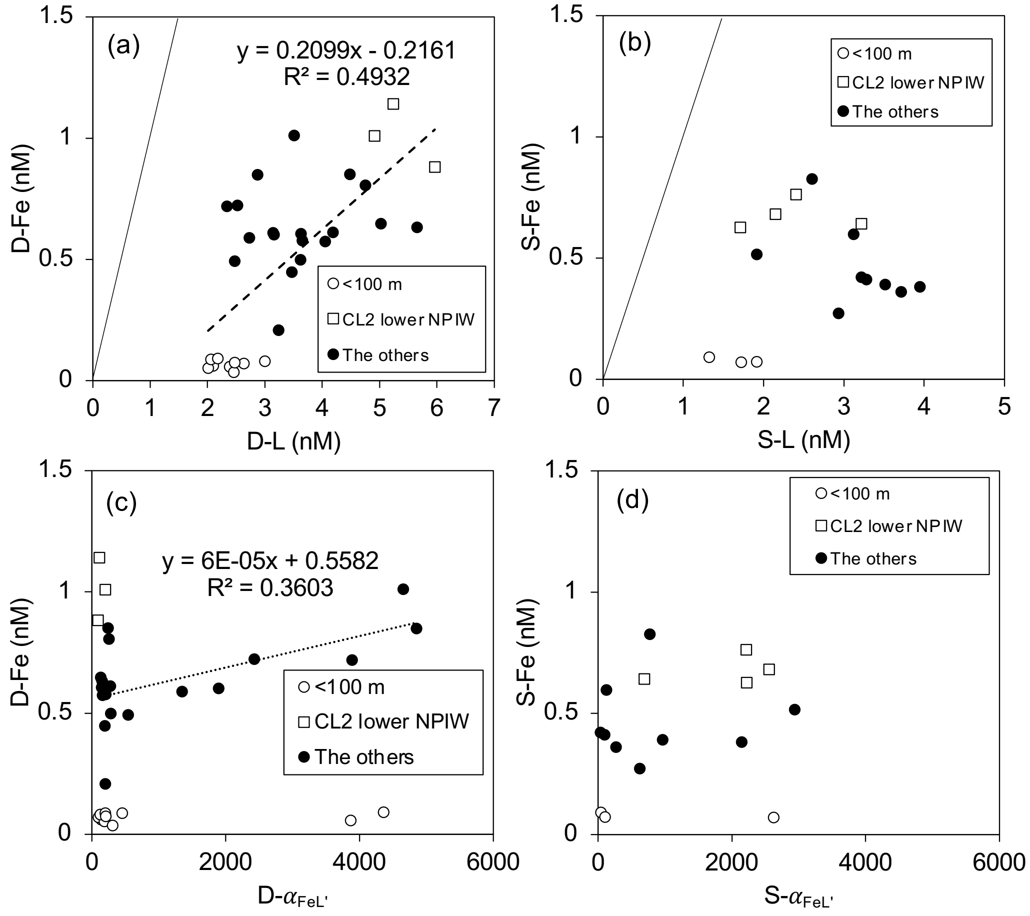

Fig. S3. Relationships between ligands parameters and Fe concentrations: (a) [D-L] vs. [D-Fe], (b) [S-L] vs. [S-Fe], (c) D-$\alpha_{FeL'}$ vs. [D-Fe] and (d) S-$\alpha_{FeL'}$ vs. [S-Fe]. The NPIW distributed below 100-m depth in this study. Straight lines in (a) and (b) represent the coincidence of Fe and ligands' concentrations. A broken line in (a) represents the regression line between [D-L] and [D-Fe] in all samples. A dot line in (c) represents the regression line between D-$\alpha_{FeL'}$ and [D-Fe] below the NPIW except for the lower NPIW density range at Stn. CL2.
